# ACCULTURATION, ALCOHOL USE, AND SLEEP AMONG LATINX ADULTS AT RISK FOR ALZHEIMER’S DISEASE AND RELATED DEMENTIAS

**DOI:** 10.1093/geroni/igad104.3160

**Published:** 2023-12-21

**Authors:** Daniel Jimenez, Shanna Burke, Timothy Hayes, Adrienne Grudzien, Accacia Russell, Gianinna Munoz Soto, Eriana Peralta, Eleni Padden

**Affiliations:** Florida International University, Miami, Florida, United States; Florida International University, Miami, Florida, United States; Florida International University, Miami, Florida, United States; Florida International University, Miami, Florida, United States; Florida International University, Miami, Florida, United States; Florida International University, Miami, Florida, United States; Florida International University, Miami, Florida, United States; Florida International University, Miami, Florida, United States

## Abstract

**Background:**

Excessive alcohol use can negatively impact sleep. In the U.S., Latinx people are most likely to engage in problematic drinking when compared to other racial/ethnic groups. Acculturation in U.S. society has been suggested as a contributing factor to problematic drinking. Excessive alcohol use and poor sleep are known risk factors for developing Alzheimer’s disease and related dementias (ADRD). Little is known about the interaction between acculturation and alcohol use on sleep outcomes in Latinx adults, especially among those at risk of ADRD. The current study explored whether acculturation to the U.S. moderated the relationship between alcohol use and sleep.

**Methods:**

A secondary analysis of a community-based sample of 113 Latinx adults, ages 40-60, was conducted. Participants had a parent diagnosed with ADRD. Measures included surveys gauging alcohol use (WHO ASSIST V.30), acculturation (Bicultural Involvement Questionnaire), subjective sleep (Pittsburgh Sleep Quality Index), and objective sleep measures using actigraphy (ActiGraph). A multiple regression analysis was performed to evaluate whether acculturation moderated the relationship between alcohol use and sleep outcomes

**Results:**

The sample was predominantly female (92%), people born outside the US (74%), with approximately 83% of participants reported lifetime alcohol use. Americanism acculturation (b= 0.30, SE= 0.21, p=0.007) and biculturalism (-b= 0.30, SE= 0.13, p=0.003) significantly moderated the relationship between alcohol use and sleep duration. Implications: Expanded, targeted research and intervention focused on sociocultural factors impacting alcohol use for Latinx people at risk of ADRD must be further explored to identify and intervene in sleep disparities occurring among this population.

